# Case Report: Multisystemic life-threatening gunshot injuries in an adult Vizsla dog

**DOI:** 10.3389/fvets.2025.1701142

**Published:** 2026-01-08

**Authors:** Bérengère Cyrielle Hadrienne Gremillet, Geoffrey Troussier, Lucia Serlenga

**Affiliations:** 1 Vet Radiology, Le Bessat, France; 2 Centre Hospitalier Veterinaire Fregis, Gentilly, France; 3 Centre Hospitalier Veterinaire Saint-Martin, Allonzier-la-Caille, France

**Keywords:** computed tomography, diagnostic imaging, dog, emergency care, gunshot, life-threatening, radiography, ultrasound

## Abstract

An 8-year-old male Vizsla dog was presented to the emergency and critical care unit due to polytraumatic injuries from a rifle shot. Multimodal diagnostic imaging revealed life-threatening injuries affecting the cardiovascular, respiratory, and gastrointestinal systems. Less severe injuries to the left kidney and retained pellets in the peritoneum and musculoskeletal system were also identified. Lesion triage and targeted treatment—including emergency stabilization, thoracotomy (subtotal pericardiectomy), and abdominal laparotomy (enterotomies and gastrotomy)—combined with tailored medical management resulted in dog’s full recovery.

## Introduction

Gunshots can cause life-threatening injuries to dogs. Of all trauma types in dogs, penetrating ballistic traumas have the highest fatality rate after blunt trauma in dogs hit by vehicles (with respective fatality rates of 17.8 and 20.2%) (1). Although gunshot injuries are reported to be rare in veterinary medicine, with an overall prevalence of less than 0.1% of all trauma types (1), geographical location and cultural factors may change this frequency (2).

The prevalence of ballistic injuries among all trauma types in dogs shows little variation between urban and rural environments, with reported rates of 0.5 and 0.6%, respectively, in one study (1). Additionally, another study found no statistically significant difference in prevalence between these environments (3). Nevertheless, geographical location and environment certainly have an impact on the lesion type and location. The type of projectile, the velocity of impact, and the type of tissue affected by the projectile depend on the circumstances surrounding the injury and the intentions of those yielding the weapons. All these parameters contribute to the nature of gunshot wounds (2, 3).

There are several types of guns that can be categorized based on their characteristics. Handguns are generally classified as low- to medium- velocity firearms, while rifles are medium- to high-velocity firearms. Shotguns are intended for killing small, fast-moving prey. However, the velocity of shotgun pellets rapidly diminishes due to their poor aerodynamic design, and the pattern of the pellets spreads out as the distance from the muzzle increases. Shotguns are designed for targets located between 25 and 35 meters away and are highly destructive at shorter ranges. This latest type of gun is often fired at dogs and cats in rural areas (4).

Initial tissue crushing and laceration are the main damage mechanisms for low-velocity missiles. High-velocity projectiles lead to two additional injury mechanisms: shock wave and cavitation. A shock wave is produced by the compression of tissue ahead of the projectile, which can damage structures that distant from the projectile’s trajectory. Cavitation is created by the projectile’s high velocity, which displaces tissue particles forward and sideways after the projectile has passed. This temporary cavity creates a vacuum that may pull dirt and debris into the wound at the projectile’s entry and exit points. In some instances, it may lead to the formation of a permanent cavity that can up to 30 times the diameter of the projectile (4).

Understanding the type of firearm used is fundamental to tailoring care and anticipating potential complications related to initial injuries. Complementary examinations aid in more accurately determining the location and extent of injuries.

This case report describes the clinical presentation, diagnostic imaging, surgical approach, and outcomes of a dog presenting with multiple high-velocity gunshot wounds and life-threatening complications.

## Case report

An 8-year-old intact male Vizsla was found lethargic with multiple pinpoint skin wounds after being missing for 1 day. The dog was immediately taken to the emergency room of the closest veterinary referral center. Physical examination revealed approximately 30 puncture wounds, predominantly on the left side of the thorax and abdomen, with minimal signs of pain on palpation. The dog was ambulatory with signs of shock: pale mucous membranes, prolonged capillary refill time, and tachycardia (heart rate = 130 bpm). The respiratory examination did not show any abnormality apart from a slightly increased respiratory rate (32 bpm). Based on these findings, a shotgun wound is suspected.

Emergency evaluation showed mild anemia (26% [reference: 37.3–61.7]) with normal total solids (65 g/L [reference: 52–82]). Complete blood count and chemistry were unremarkable apart for mild non-regenerative anemia (reticulocytes 42.7 × 109/μL [reference: 10–110], MCV 63.3 fL [reference: 61.6–73.5], MCHC 34.9 g/dL [reference: 32.0–37.9]) and increased lactate (3.6 mmol/L [reference: 0–2.0]). Thoracic point-of-care ultrasound (T-POCUS) revealed bilateral loss of glide sign in the dorsal thoracic region when the dog was standing, consistent with mild pneumothorax, and several B-lines in the whole pulmonary area, consistent with multifocal pulmonary consolidations. At this stage, multifocal pulmonary consolidations were most consistent with poor pulmonary insufflation (secondary to the pneumothorax), pulmonary hemorrhages, or, less likely, emerging pulmonary infection. A small amount of echoic fluid between the thoracic walls and the lung lobe margins also indicated mild pleural effusion. Total solids and hematocrit in pleural effusion were measured at 60 g/L and 30%, respectively, consistent with an active hemothorax. Abdominal point-of-care ultrasound (A-POCUS) showed no signs of effusion at the time of presentation.

Initial care was conservative medical management with intravenous fluid therapy (Ringer’s lactate at 20 mL/kg IV for 20 min, then 3 mL/kg/h),^1^ antibiotics (ampicillin and sulbactam^2^ at 20 mg/kg IV every 8 h), and morphine^3^ (0.3 mg/kg IV every 4 h). After 24 h of initial medical management, melena was noted. Physical examination revealed tachycardia (144 bpm), pale mucous membranes, and weak femoral pulses. The hematocrit dropped to 16% with a total solids of 36 g/L. A hemorrhagic shock was suspected, prompting a blood transfusion. The dog received 20 mL/kg of refrigerated whole blood over 4 h. The transfusion was uneventful, normalized the patient’s vitals, and brought up the PCV/TP to 24%/50 g/L. Rechecked thoracic and abdominal POCUS were comparable to the previous day.

Diagnostic imaging was used for further evaluation. The dog received morphine (0.3 mg/kg IV) and was anesthetized with midazolam (0.3 mg/kg IV), propofol (1 mg/kg IV), and isoflurane during the CT scan. Thoracic radiographs^4^ within 2 h after admission, and thoracic and abdominal computed tomography^5^ 12 h after admission, identified and localized multiple pellets and associated soft tissue injuries.

Multiple subcutaneous and muscle-embedded pellets were identified in the thoracic limbs and in the thoracic and abdominal walls. Peripheral soft tissues and bones were unremarkable.

A mild bilateral asymmetric pneumohydrothorax was confirmed on CT scan, more severe in the left hemithorax, resulting in lung lobe retraction and decreased volume. Peripheral parenchymal bands were present in all lung lobes, and an area of pulmonary consolidation was observed in the ventral part of the caudal subsegment of the left cranial lung lobe. The main differentials for these pulmonary injuries include poor pulmonary insufflation and atelectasis.

**Figure 1:**
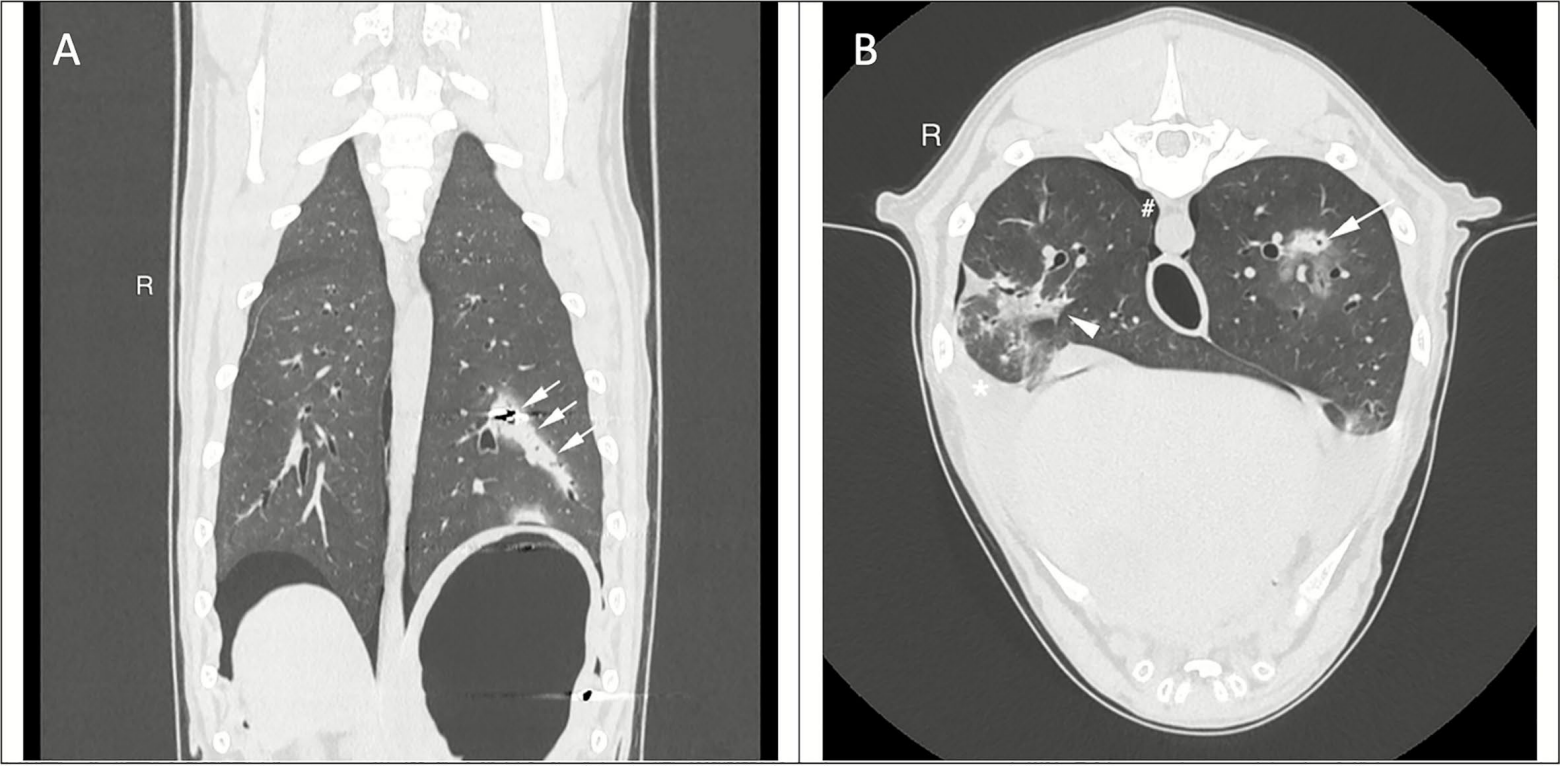
CT acquisition of the thorax. **(A)** Dorsal reconstruction, lung window, a hyperattenuating linear tract (arrows) extends from the caudolateral periphery of the lobe to the pellet location. **(B)** Transverse reconstruction, lung window, pneumothorax, and pleural effusion cause pulmonary atelectasis in the right caudal lung lobe (arrowhead). The linear tract in the left caudal lung lobe has a round and well-delineated transverse section (arrow).

A pellet was identified in the middle of the left caudal lung lobe and associated with a well-defined hyperattenuating linear tract measuring 8 × 69 mm (width x length) and multiple punctate gas foci extending to the caudo-lateral periphery of the lobe (Figures 1A, B).

There was a pellet along the left diaphragmatic cupola, in contact with both the lung and liver. A small area of consolidated lung parenchyma adjacent to the pellet could be identified on CT scan, but not on radiographs. The adjacent hepatic parenchyma was unremarkable.

On the CT scan, three pellets were identified at the cranial aspect of the right ventricle, in the pericardium or in the myocardium, and one pellet was identified between the main pulmonary artery and the aorta. There was no evidence of associated pericardial effusion, distortion of the cardiac chambers, or great vessels.

Six pellets were identified along the greater curvature of the stomach, at the level of the gastric body and pylorus. There was no evidence of adjacent peritoneal steatitis, gas, or effusion.

A pellet was also identified between the urinary bladder and the colon. The exact location of the pellet remained unclear due to the proximity and inherent thin-wall thickness of these organs.

A pellet was identified in abdominal fat along a segment of the small intestine, medial to the left kidney. On post-contrast images, a lateromedial hypoattenuating linear tract measuring 3 × 50 mm (width × length) was visible across the caudal pole of this kidney, immediately ventral to the renal pelvis. A slight subcapsular effusion was present adjacent to this lesion, surrounding the caudal pole of the kidney.

A small pellet fragment was visible in the middle of the right abdomen, along an irregular segment of the small intestine. Adjacent steatitis associated with two pinpoint gas foci surrounded by the irregular intestinal wall. A few additional small free peritoneal gas bubbles were identified dorsal to the right pancreatic lobe.

The main differential diagnosis for peritoneal injuries was septic peritonitis and pneumoperitoneum due to the perforation of a segment of the small intestine by a pellet. Less consideration was given to focal inflammation and gas formation secondary to the passage of a high-velocity pellet. Gastric ulcers and luminal hemorrhage due to direct injury by multiple pellets were included in the differential diagnosis to explain the anemia. Gastric perforation was considered less likely. Despite the absence of any signs of hemorrhagic changes around the heart and great vessels of the thorax, cardiovascular injuries were considered an additional differential diagnosis for anemia.

**Figure 2:**
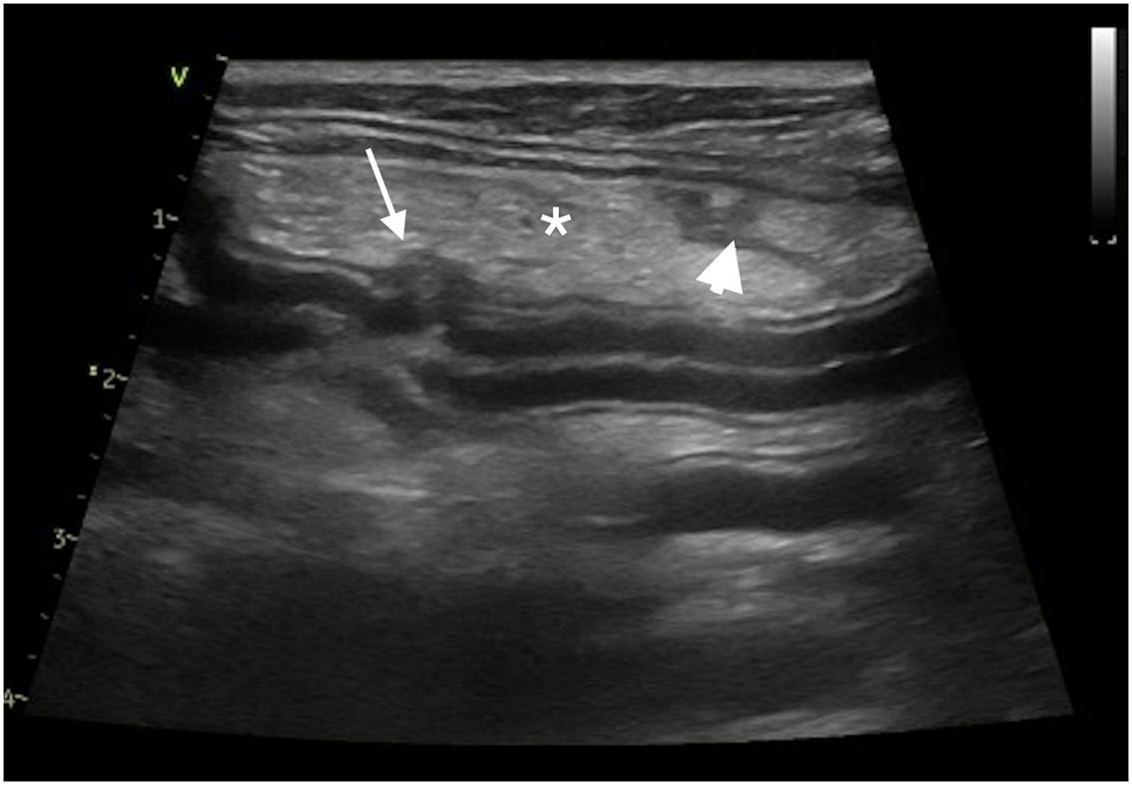
Abdominal ultrasound. A focal disruption of a jejunal segment is identified (arrow) with adjacent steatitis (asterisk) and a small amount of peritoneal effusion (arrowhead).

Abdominal ultrasound^6^ confirmed focal disruption of the jejunal wall with adjacent steatitis and scant peritoneal effusion (Figure 2). There was no evidence of gastric perforation.

Standard trans-thoracic echocardiography^7^ showed a small amount of anechoic pericardial effusion. A cluster of pellets was identified in the pericardium near the apex of the right ventricular wall without evidence of an adjacent myocardial lesion. A second pellet was identified between the aorta and the main pulmonary artery, with both vessels being unremarkable. There was no evidence of cardiac rhythm or blood flow disorders.

Considering the patient’s clinical signs and imaging results, the primary concerns were intestinal perforation leading to pneumoperitoneum and peritonitis; pulmonary contusions and pneumothorax resulting in pneumohemothorax; and a mild pericardial effusion associated with three pericardial, along with another pellet situated near the aorta and main pulmonary artery. The renal and gastric lesions, as well as other peritoneal pellets, were deemed less clinically significant. Pellets located in the musculoskeletal tissues and subcutaneous space were regarded as minor findings.

A thoracotomy was performed through the fourth left intercostal space, with a mild hemothorax present. Except for a visible pellet tract in the left cranial and caudal lung lobes, all lung lobes were inflated and had a normal macroscopic appearance. The three pericardial pellets were identified and prompted subtotal pericardiectomy. There was no evidence of cardiac injuries, and the most deeply located pellet could not be visualized between the great vessels at the base of the heart. A small-bore thoracostomy tube^8^ was placed on the left thoracic wall at the end of the surgery, allowing intermittent active drainage post-operatively. The drain was suctioned twice daily and removed 3 days later, once the production was less than 2 mL/kg/day.

For the laparotomy, a ventral midline approach was chosen. Two jejunal perforations and hepatic wounds were identified. Given the small size of the intestinal injuries and adequate vascularization, jejunal enterotomies and omentalization were performed. The hepatic injuries did not need to be surgically addressed based on their macroscopic appearance at the time of surgery. There was no evidence of gastric perforation. A peri-operative gastric endoscopy showed large and deep gastric ulcers at the greater curvature of the stomach. Based on these findings and previous identification of multiple pellets in the gastric wall, a gastrotomy and partial gastrectomy were elected to prevent ulcer-related complications and to avoid future lead intoxication. In the context of septic peritonitis, an abdominal drain was placed at the end of the laparotomy.

Post-operatively, antibiotic therapy was continued and combined with omeprazole (1 mg/kg PO every 12 h),^9^ smectite (two portions every 12 h),^10^ sucralfate (1 gr PO every 24 h),^11^ maropitant (1 mg/kg IV every 24 h),^12^ and metoclopramide (0.08 mg/kg/h IV).^13^ The abdominal drain was suctioned twice daily, and the amount of effusion was measured on a daily basis. Daily cytology analysis of the peritoneal effusion was performed to ensure the absence of bacteria. The drain was removed 2 days later, once the daily amount was less than 2 mL/kg/day and no bacteria were observed. Neither signs of hypoalbuminemia, hypoglycemia, hypotension, nor tachycardia were noticed in the post-operative period.

Three days after the surgery, the dog started to cough with no signs of dyspnea. Thoracic radiographs revealed a ventral alveolar opacification in the caudal part of the left cranial lung lobe and in the left caudal lung lobe. The consolidation surrounding the pellet in the left caudal lung lobe appeared moderately enlarged and increased in opacity in comparison to the initial radiographic study. At this stage, differential diagnosis included a pulmonary infection due to inoculation by direct pellet penetration, temporary cavitation, or hematogenous spread. Other differentials included parenchymal lung necrosis, localized pulmonary hemorrhage, aspiration pneumonia, or sterile pneumonitis. Treatment options included lung lobectomy to remove the pellet and pulmonary injuries or medical management. The owners opted for medical management. Additional antibiotic therapy (trimethoprim/sulfamethoxazole^14^ (TMPS) at 15 mg/kg PO every 12 h) was initiated in combination with the amoxicillin/clavulanic^15^ acid previously implemented. During the following days, the cough improved. The size of the alveolar opacifications markedly decreased on repeated radiographs 3 days later, consistent with a positive response to treatment and confirming the presumptive diagnosis of lobar pneumonia and local infection around the dorsally located pellet.

After 2 weeks of hospitalization, the dog showed a good clinical response and started to gain weight. There were no more signs of gastrointestinal bleeding, and respiratory signs returned to normal.

The renal injury and subcapsular effusion were monitored with ultrasound during hospitalization of the patient and remained stable. Biochemical parameters remained unremarkable between initial admission and discharge of the patient, consistent with minimal to absent disturbance of renal function.

The patient was discharged from the hospital with one more week of amoxicillin/clavulanic acid (15 mg/kg PO every 12 h) and trimethoprim/sulfamethoxazole (15 mg/kg PO every 12 h). Follow-up radiographs at the end of the treatment showed complete resolution of the pulmonary parenchymal injuries.

Three months after the initial presentation, a phone contact with the owner informed us that the dog is alive and doing well.

## Discussion

This paper describes a variety of rifle shot injuries in a dog and successful critical care management. Triage of injuries is a key point in a polytraumatized patient, both to give an early vital prognosis and to adapt treatment. The animal trauma triage score (ATT score), a triage scoring system, has been suggested for gunshot injuries in cats and dogs to predict short-term outcomes and optimize case management (2). Although there was no ATT score initially calculated for this patient, it is suspected that the score improved over hospitalization. Critical care management included reestablishment of a normal perfusion with fluid therapy and whole blood transfusion, followed by treatment of cardiac, respiratory, and gastrointestinal life-threatening injuries (thoracotomy and abdominal laparotomy). Surgical exploration was elected since this is recommended for any penetrating injury affecting the thoracic or abdominal cavities (5–7). The owner’s awareness of the guarded prognosis, cost of procedures, and potential complications was fundamental in this critical patient. Immediate, comprehensive, and efficient patient management was possible and proved to be successful.

Thoracic wounds are reported to be the most common injuries in animals with gunshot injuries after limb injuries. Hemothorax and pneumothorax are often reported in association with penetrating thoracic injuries and may be life-threatening. Surgical exploration is highly recommended in some studies (5). Although other studies recommend it only if there is perforation of vital structures or if medical management, for instance thoracostomy tube alone, is not sufficient (2, 3, 8). Radiography is a rapidly available and minimally invasive imaging modality that may be useful to confirm involvement of the thoracic cavity and potential impairment of vital structures such as the lungs and heart. Radiographs were justified for the initial assessment of this patient and adapted to its out-of-hours arrival at the hospital. A CT scan under general anesthesia was scheduled for the following day to allow time for clinical stabilization of the patient. A safe and good quality CT scan allowed clarification of the location of the thoracic pellets and allowed further evaluation of related pulmonary and cardiovascular injuries. Four pellets were of main concern as they were close to the right ventricle and to the great vessels at the base of the heart. The most common complications of cardiac pellets include pericarditis, endocarditis, and aortic or right ventricle fistula (9). Echocardiography is the modality of choice for assessing cardiac structures and function in veterinary patients, as it allows dynamic evaluation with good soft tissue contrast and detail. It is complementary to static imaging modalities such as radiography and computed tomography, which may be affected by cardiac motion and breathing. Although electrocardiogram-gated cardiac CT (ECG-gated CCT) is a promising alternative to echocardiography in human medicine (10), it is a relatively new technology in veterinary medicine, which is rarely available outside university hospitals. In veterinary patients, ECG-gated CCT also requires sedation that may impact cardiac function (11). In this patient, the cardiac chambers and vessels were not impaired by the pellets. The pellets might have gone around rather than through these important cardiovascular structures. Alternatively, the small size of the projectiles and the relative elasticity of cardiovascular structures could have allowed rapid sealing of the point of entrance of the pellets, preventing major local hemorrhage or aneurysm. Two pellets were identified in the lungs, associated with parenchymal consolidation, likely due to local hemorrhage on the pellet tracks, and pneumohemothorax. There is a small risk of embolization when a projectile passes through a blood vessel, and it is often initially asymptomatic (12). Migration of a projectile to a pulmonary artery or to peripheral capillaries can lead to thrombosis and ischemia (8, 13). In this patient, subtotal pericardiectomy was elected to prevent further cardiac complications and to treat the hemopericardium. Thoracotomy also allowed further evaluation of the pulmonary injuries. Scar tissues in different lung lobes were visible but did not impair pulmonary function, and there was no indication for lung lobectomy at this stage.

According to Bebchuk and Harari (4), all gunshot injuries are infected. Indeed, the pellet or cavitation phenomenon may bring hair and skin debris into the wound and secondary lead to infection. *Staphylococcus* spp. are assumed to be the primary sources of infection. Broad-spectrum bactericidal antimicrobials are recommended. Although there is a large choice of antibiotics available in human medicine, cephalosporines or penicillin in combination with an aminoglycoside antibiotic are reported to be a good choice for initial treatment (4). In this patient, a broad spectrum of antibiotics (ampicillin/sulbactam) was initially administered. Pulmonary infection during hospitalization was an unexpected complication, but the eventual combination of Ampicillin/Sulbactam with TMPS proved to be sufficient. Other antimicrobials may be available both in veterinary and human medicine, such as enrofloxacin; however, based on regional restrictions about the use of antimicrobials, enrofloxacin was not a suitable antibiotic. Enrofloxacin can be chosen if a specific bacterial culture shows it is the only antimicrobial drug that can be used. A transtracheal wash or a bronchoalveolar lavage might have been interesting to obtain culture samples prior to any treatment; however, the patient’s critical condition upon admission prevented these tests from being performed. Additionally, bacterial culture and sensitivity would have taken several days to obtain a result. TMPS is active against a broad spectrum of Gram-positive bacteria, including some strains of methicillin-resistant *Staphylococcus* spp. (MRS), and a broad spectrum of Gram-negative bacteria, which may be complementary to ampicillin/sulbactam, active against Gram-positive bacteria except for MRS, some Gram-negative bacteria, and anaerobes (www.msdmanuals.com) (14). Although no bacterial culture can prove the exact origin of the infection, an MRS might have caused the infection when getting inside the body with pellets. Indeed, MRS *pseudintermedius* (MRSP) is a bacterium that usually lives harmlessly on the skin of canine patients, but can cause infection when present inside the body. Some Gram-negative bacteria commonly found in dogs with pneumonia, such as *Klebsiella pneumonia* and *Escherichia coli* (15), may also be resistant to ampicillin/sulbactam and sensitive to TMPS.

Peritonitis and pneumoperitoneum secondary to an intestinal perforation are possible complications of abdominal gunshot injuries. Gastrointestinal injuries often remain unnoticed until peritonitis occurs. Peritonitis should be a differential diagnosis in dogs with lethargy, depression, collapse, diarrhea, vomiting, or shock (16). In this patient, melena and anemia were additional findings suggestive of gastrointestinal injuries. Although computed tomographic examination allowed extensive evaluation of the abdominal cavity and walls and enabled the targeting of injuries related to the gunshot, it lacks contrast detail when it comes to gastrointestinal walls or intrinsic soft tissue structures. Ultrasound examination is the modality of choice for further abdominal evaluation. In association with gastroscopy, it allowed further evaluation of gastric ulcers and small intestinal perforations. The cause of the perforations remained unclear; the main differentials included direct pellet trauma or shock waves. As for the gastric ulcers, the most likely etiology is blood supply impairment and tissue reaction secondary to the wall penetration of multiple pellets at the same site. Indeed, the wadding of shotgun pellets can produce a reactive tissue response when embedded in the wound (4). There was no histopathology in the removed tissue, but infection (by MRSP or another type of bacteria) could also have contributed to the injuries. The presence of pre-existing ulcers was considered less likely, as the parietal injuries were centered on pellets embedded in the gastric wall. In any case, exploratory laparotomy with gastrotomy and enterotomies allowed the successful management of these injuries.

Gunshot musculoskeletal injuries are often a concern for direct traumatic injuries affecting the spine or other parts of the skeleton. In this patient, there was no evidence of bone fracture or spinal injury. Pellets in the musculoskeletal system are often non-toxic as they are sealed in a fibrous capsule. Toxicity may occur if there is contact with body fluids such as synovial, pleural, or cerebrospinal fluid or with the gastrointestinal tract. An increased number of pellets and an increased surface-area ratio (such as in fragmented pellets) also increase the risk of intoxication (17). A rather recent study has described lead intoxication in dogs fed with trimmings of lead-shot game (18). Lead absorption can cause acute or chronic intoxication with rather non-specific neurological and gastrointestinal disorders, and sometimes bone or renal disorders. Blood analysis can show mild hypochromic microcytic anemia and an increased whole blood lead level (19). In this patient, musculoskeletal pellets were left in place. Although there was no evidence of lead intoxication on blood analysis, exploratory laparotomy for the observed gastric ulcers allowed the preventive removal of all gastric pellets. The dog was clinically healthy 3 months after the accident, and there has not been any clinical evidence of lead intoxication.

## Conclusion

This case report demonstrates how multimodal diagnostic workup can be used to prioritize treatment options and prevent complications associated with polytraumatic injuries from rifle shots.

## Data Availability

The raw data supporting the conclusions of this article will be made available by the authors, without undue reservation.

## Footnotes

1 Ringer lactate, B Braun Medical SA, Barcelone, Spain.

2 Unacim 1 g Pzizer, Paris, France.

3 Morphine AGUETTANT, Lyon, France.

4 DRX compass Carestream, Canestream health Inc., New York, USA. 70 KVp et 6,3 mAs.

5 Canon Aquillon lightning, Canon medical systeme Europe B. V.,Zoetermeer, The Netherlands. Pre and post contrast acquisitions were obtained using 600 mg/kg of Iohexol IV. Soft tissue reconstruction (1 slice thickness) and pulmonary reconstruction (1 mm slice thickness) algorithms were used for interpretation. The parametres used was a 120 Kv and 30 mA gantry rotation time à 0.75 s and a collimation of 0.5 × 80 mm.

6 Philips’s affinity 50, Philips Ultrasound Inc., Washington, USA. C8–5 micro convex probe on dorsal recumbency.

7 Philips’s affinity, Philips Ultrasound Inc., Washington, USA. Fifty machine and S 8–3 cardiac probe on lateral recumbency.

8 Mila Chest Tube - 14Fr × 38 cm, MILA International Inc., Kentucky, USA.

9 Omeprazole 20 mg, Cristers, Suresnes, France.

10 Smectite, Vetinnov SAS, laboratoire Osalia, Paris, France.

11 Keal EXOD, Saint Cloud, France.

12 Prevomax 10 mg/mL, Dechra Veterinary Products SAS, Montigny-le-bretonneux, France.

13 Metomotyl, Dechra Veterinary Products SAS, Montigny–le–Bretonneux, France.

14 Sultrian, Ceva santé Animale, Libourne, France.

15 Kesium, Ceva Santé Animale, Libourne, France.
