# Supplementary material for: Case Report: Multisystemic life-threatening gunshot injuries in an adult Vizsla dog
Source: Front Vet Sci. 2026 Jan 8;12:1701142. doi: 10.3389/fvets.2025.1701142 (PMC12824419; doi:10.3389/fvets.2025.1701142)
Supplement: Supplementary file 1 [file Data_Sheet_1.DOCX]

**Supplementary Figures**

*
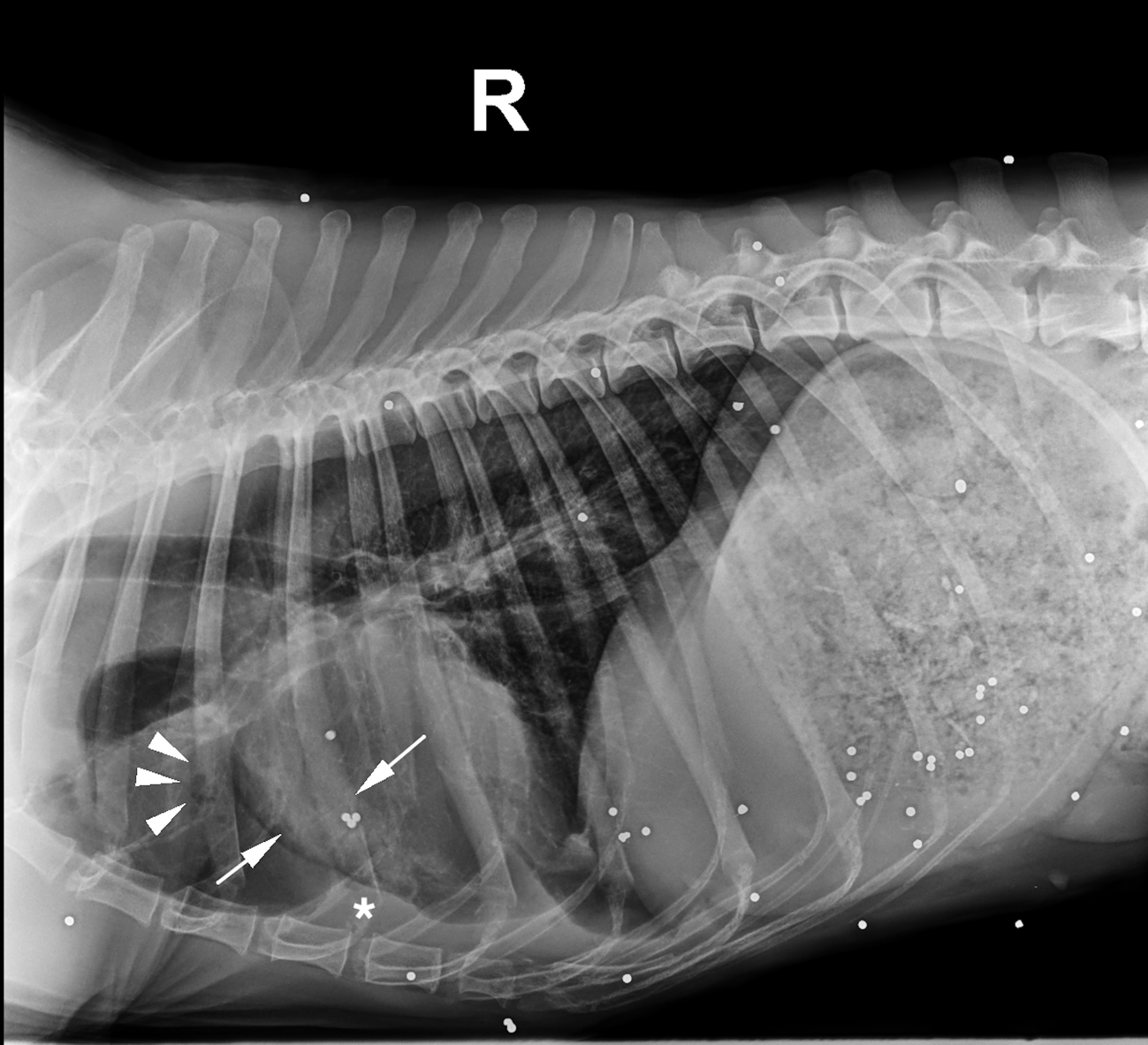
*

*Figure 3 Thoracic radiograph at presentation, right lateral view. The multiple pinpoint opacities are consistent with pellets. A moderate amount of pleural effusion (asterisk) is noted ventrally, and coalescing gas bubbles are identified cranially (arrowheads). An ill-delineated pulmonary consolidation is visible in the caudal subsegment of the left cranial lung lobe (arrows).*

*
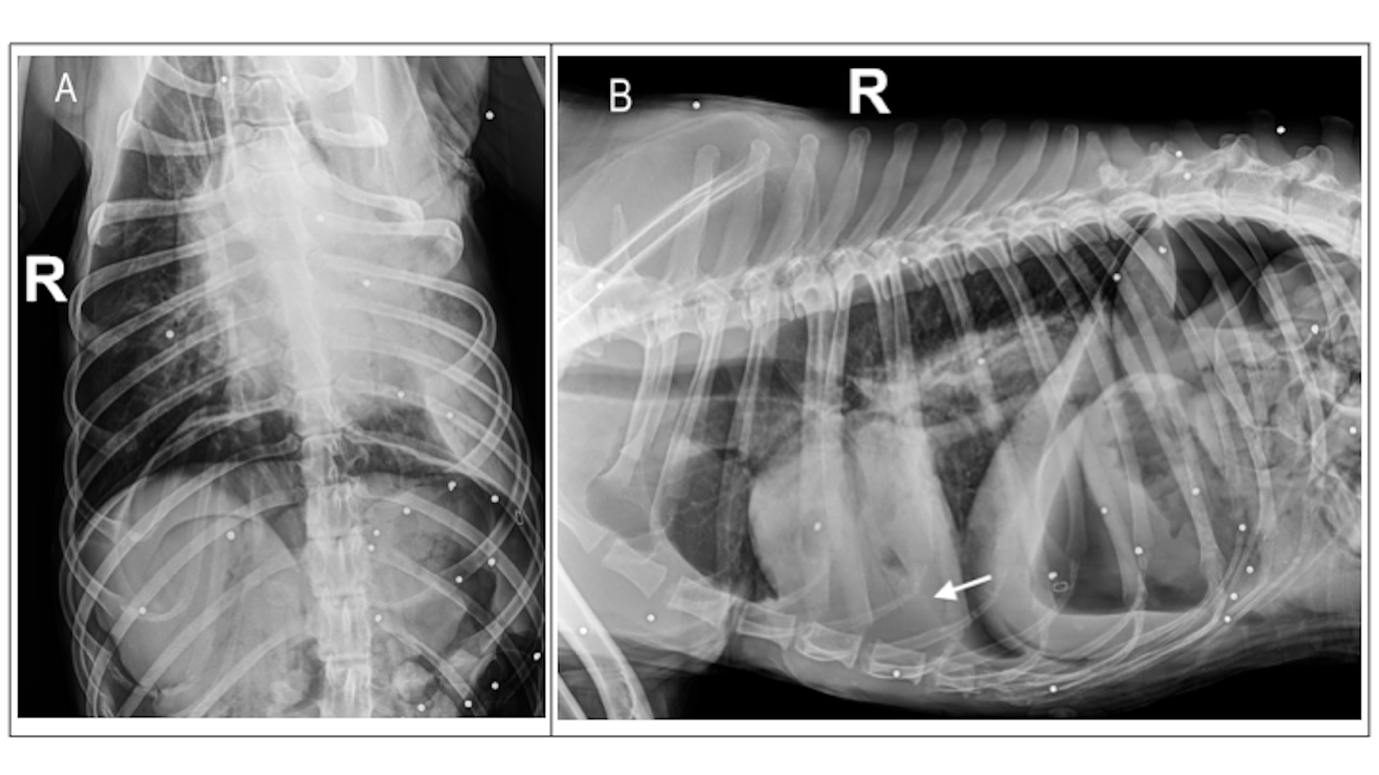
*

*Figure 4 Thoracic radiograph, three days post-operative (****A****) Ventro-dorsal view, the consolidation in the left caudal lung lobe is markedly enlarged compared to Figure 3. (****B****) Right lateral view, the ventral consolidation of the caudal subsegment of the left cranial lung lobe is enlarged. There is a well-delineated lobar sign between the left cranial and left caudal lung lobes (arrow). Note that the previously described pericardial and gastric pellets have been removed during the surgery and are no longer visible.*

*
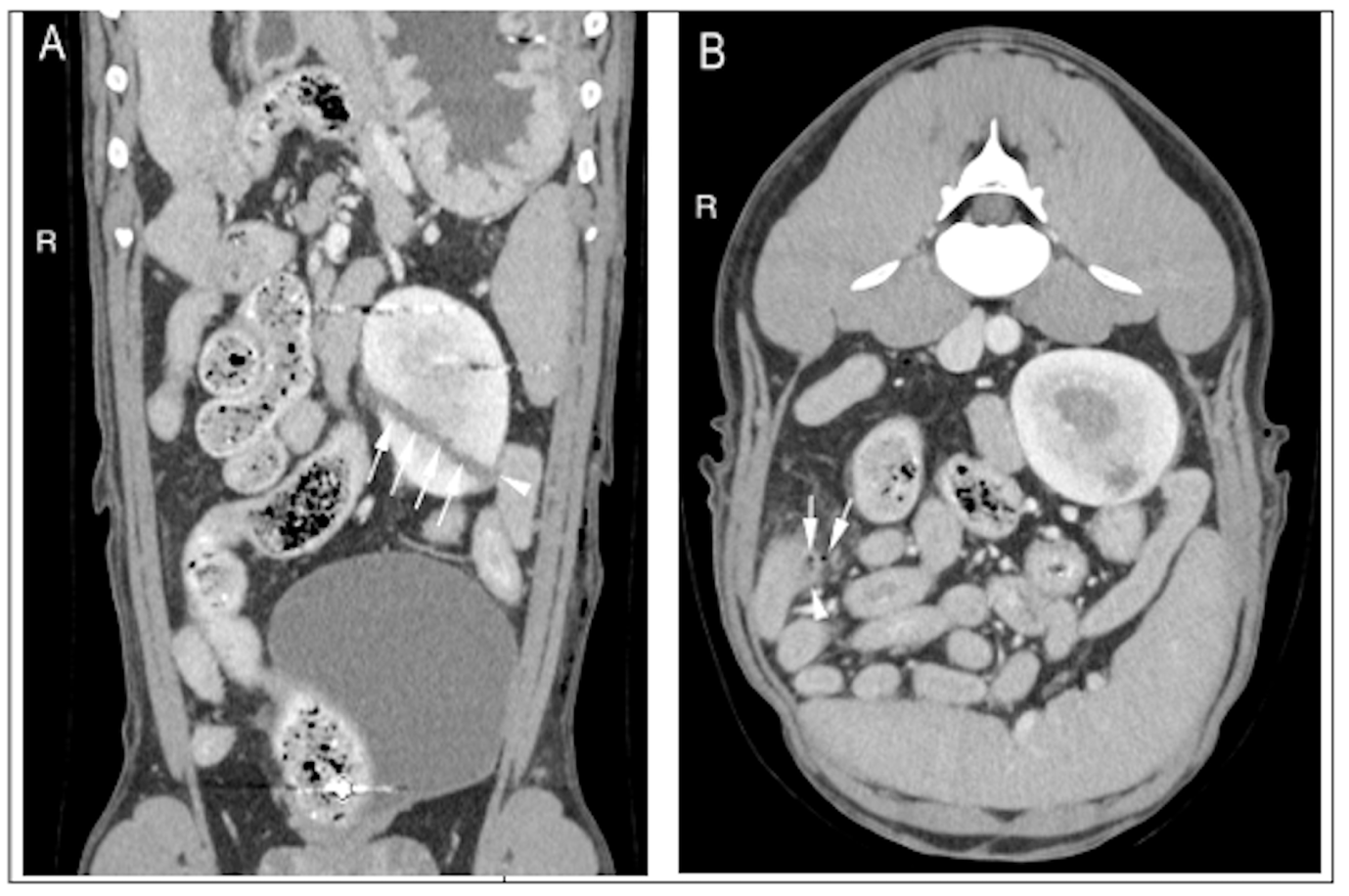
*

*Figure 5 Post-contrast CT acquisition of the abdomen. (****A****) Dorsal reconstruction at the level of the left kidney, soft tissue window, a linear hypoattenuating tract through the caudal pole of the kidney is noticed (arrows), associated with a mild amount of subcapsular effusion (arrowhead). (****B****)Transverse reconstruction, soft tissue window, at the level of an abnormal small intestinal segment. The intestinal wall is irregular (arrowhead). There are focal adjacent steatitis and two pinpoint gas foci (arrows). Note the cross section of the well-delineated hypoattenuating tract in the ventral aspect of the left kidney.*

*
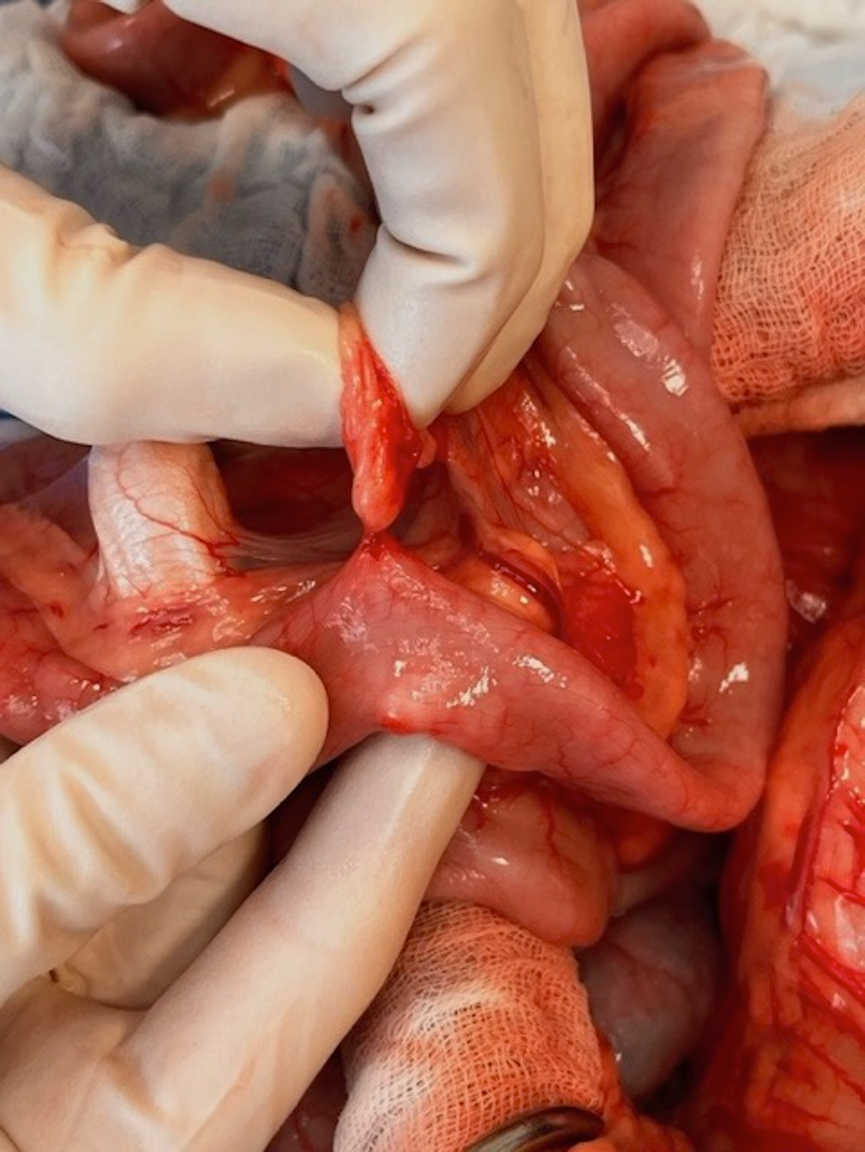
*

*Figure 6 Photo taken during laparotomy showing intestinal perforation and mesenteric patch.*
